# Supplementary material for: Enhanced JBrowse plugins for epigenomics data visualization
Source: BMC Bioinformatics. 2018 Apr 25;19:159. doi: 10.1186/s12859-018-2160-z (PMC5921565; doi:10.1186/s12859-018-2160-z)
Supplement: Supplementary file 1 — Figure S1. Supplementary methods. (PDF 100 kb) [file 12859_2018_2160_MOESM1_ESM.pdf]

SUPPLEMENTARY FIGURE

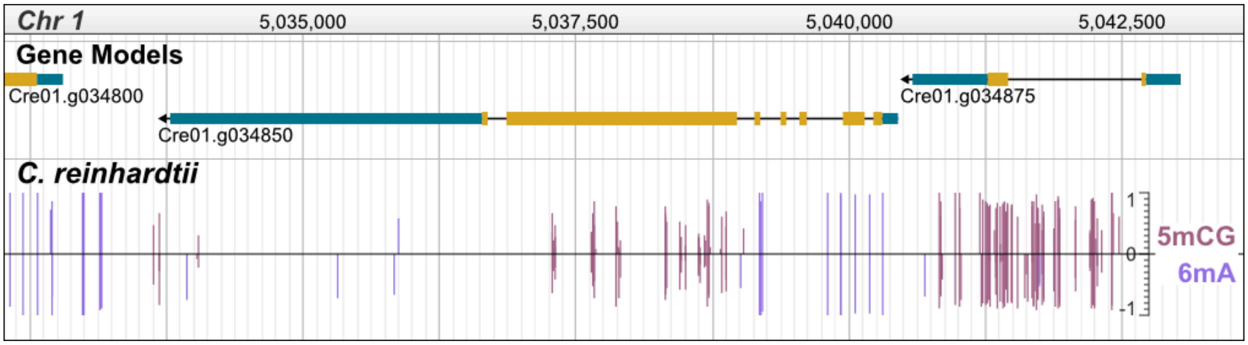

Fig. S1. Base modifications of *C. reinhardtii*. Viewing 5mC in the CG sequence context and 6mA in *C. reinhardtii* with the plugin. Height and direction of bar indicates methylation level and strand, respectively. Bars are colored by 5mCG and 6mA.

SUPPLEMENTARY METHODS

Genomes.

Arabidopsis thaliana: Reference sequence, gene models, and transposable element models were from the TAIR10 genome (https://www.arabidopsis.org).

Chlamydomonas reinhardtii: Reference sequence (v5.0) and gene models (v5.5) were from Phytozome 12 (https://phytozome.jgi.doe.gov/pz/portal.html).

Fig. 1: *A. thaliana* 5-methylcytosine.

Samples were previously published [1] and raw reads were downloaded SRR342383, SRR342378, and SRR342381 for sample 1, sample 2, and sample 3, respectively. Briefly reads were processed and aligned as described in Schultz *et al.* [2]. Reads were trimmed using Cutadapt v1.3 [3] with parameters "-a AGATCGGAAGAGCTCGTATGCC -m 30 -q 10". Reads were mapped to the TAIR10 genome using Bowtie v1.1.0 [4] with parameters "-k 1 -m 1 --chunkmbs 3072 --best --strata -o 4 -e 80 -l 20 -n 0". PCR duplicated were removed using Samtools v1.2 [5]. Only uniquely mapped, non-clonal reads were retained.

Resulting output files were converted to BigWig format using custom scripts available with the plugin. Methylated cytosines were determined as described in [2]. At each methylated cytosine, weighted methylation level [6] was computed as mC / (mC + uC) where mC is the number of methylated reads and uC is the number of unmethylated reads at the position. Positions were split into separate bedGraph files based on sequence context and files were converted using bedGraphToBigWig [7].

Fig. 2: *A. thaliana* small RNAs.

This sample was previously published [8] and raw reads were downloaded from SRR1266859. Reads were trimmed using Cutadapt v1.9.dev1 [3] with parameters "-a TGGAATTCTCGGGTGCCAAGGAACTCCAGT -g TGTGTTCTCAGGTCGCCCCTG -m 18 -M 30". Reads were then mapped to the TAIR10 genome using Bowtie v1.1.1 [4]

with parameters “--phred33-quals -n 0 -l 18 -M 1 --best”. Samtools v1.2 [5] was used for subsequent file conversion.

Fig. 3: *A. thaliana* stranded coverage and sequence motif density.

Sample 1 methylation coverage was computed using only the uniquely mapped, non-clonal reads processed as described above in Fig. 1. Resulting BAM file was converted to BigWig format using custom scripts available with the plugin. Briefly, using the genomecov module from bedtools v2.24.0 [9], strand-specific coverage was found using the parameters “-bga -strand +” for the positive strand coverage and “-bga -strand -” for the negative strand coverage. The resulting bedGraph files were converted to BigWig format with bedGraphToBigWig [7].

CN dinucleotide (CA, CC, CG, and CT) motif density was computed using sliding windows with window size 100 base-pair (bp) and step size 10 bp. Using only the forward strand, density of the window was computed as nCN / nNN where nCN is the number of CN dinucleotides in the window and nNN is the number of dinucleotides in the window.

Figure S1: Base modifications in *C. reinhardtii*.

For 5-methylcytosine, raw reads were downloaded from SRR2022686. Paired-end reads were mapped as described in Schultz *et al.* [2]. Reads were trimmed using Cutadapt v1.14 [3] and mapped using Bowtie2 v2.2.9 [10]. PCR duplicates were removed using Picard tools v2.4.1 [11]. Only uniquely mapped, non-clonal reads were retained. Samtools v1.3.1 [5] was used for additional file conversions. Methylated reads and methylation level were determined as described above in Fig. 1.

For 6-methyladenine, processed WT data was downloaded from GSE68860. The “frac” attribute of each reported 6mA position was used to indicate methylation level.

REFERENCES

1. Schmitz RJ, Schultz MD, Lewsey MG, O’Malley RC, Urich MA, Libiger O et al. Transgenerational epigenetic instability is a source of novel methylation variants. Science. 2011; doi:DOI: 10.1126/science.1212959.
2. Schultz MD, He Y, Whitaker JW, Hariharan M, Mukamel EA, Leung D et al. Human body epigenome maps reveal noncanonical DNA methylation variation. Nature. 2015 Jul 9; doi:10.1038/nature14465.
3. Martin M. Cutadapt removes adapter sequences from high-throughput sequencing reads. EMBnet journal. 2011; 17: pp. 10-12.
4. Langmead B, Trapnell C, Pop M, Salzberg SL. Ultrafast and memory-efficient alignment of short DNA sequences to the human genome. Genome biology. 2009; 10: 1.
5. Li H, Handsaker B, Wysoker A, Fennell T, Ruan J, Homer N et al. The sequence alignment/map format and SAMtools. Bioinformatics. 2009; doi:10.1093/bioinformatics/btp352.
6. Schultz MD, Schmitz RJ, Ecker JR. ‘Leveling’ the playing field for analyses of

single-base resolution DNA methylomes. Trends Genet. 2012 Dec; doi:10.1016/j.tig.2012.10.012.

7. Kent WJ, Zweig AS, Barber G, Hinrichs AS, Karolchik D. BigWig and BigBed: enabling browsing of large distributed datasets. Bioinformatics. 2010 Sep 1; doi:10.1093/bioinformatics/btq351.
8. Li S, Vandivier LE, Tu B, Gao L, Won SY, Li S et al. Detection of Pol IV/RDR2-dependent transcripts at the genomic scale in Arabidopsis reveals features and regulation of siRNA biogenesis. Genome research. 2015; 25: 235-245.
9. Quinlan AR, Hall IM. BEDTools: a flexible suite of utilities for comparing genomic features. Bioinformatics. 2010; 26: 841-842.
10. Langmead B, Salzberg SL. Fast gapped-read alignment with Bowtie 2. Nat Methods. 2012 Apr; doi:10.1038/nmeth.1923.
11. Broad Institute. Picard. https://broadinstitute.github.io/picard/index.html. Accessed 27 May 2016.
